# Guide Me in Analysis: A Framework for Guidance Designers

**DOI:** 10.1111/cgf.14017

**Published:** 2020-05-16

**Authors:** Davide Ceneda, Natalia Andrienko, Gennady Andrienko, Theresia Gschwandtner, Silvia Miksch, Nikolaus Piccolotto, Tobias Schreck, Marc Streit, Josef Suschnigg, Christian Tominski

**Affiliations:** ^1^ Vienna University of Technology Austria; ^2^ Fraunhofer Institute IAIS Germany; ^3^ the University London UK; ^4^ TU Graz Austria; ^5^ Johannes Kepler University Linz; ^6^ Pro2Future Competence Center and AVL List GmbH Graz Austria; ^7^ University of Rostock Germany

**Keywords:** Visual Analytics, Visualization, User Interface Design, Interaction, Information Visualization, Guidance

## Abstract

Guidance is an emerging topic in the field of visual analytics. Guidance can support users in pursuing their analytical goals more efficiently and help in making the analysis successful. However, it is not clear how guidance approaches should be designed and what specific factors should be considered for effective support. In this paper, we approach this problem from the perspective of guidance designers. We present a framework comprising requirements and a set of specific phases designers should go through when designing guidance for visual analytics. We relate this process with a set of quality criteria we aim to support with our framework, that are necessary for obtaining a suitable and effective guidance solution. To demonstrate the practical usability of our methodology, we apply our framework to the design of guidance in three analysis scenarios and a design walk‐through session. Moreover, we list the emerging challenges and report how the framework can be used to design guidance solutions that mitigate these issues.

## Graphical abstract

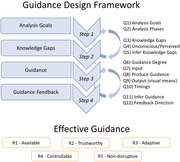

## 1 Introduction

Visual analytics (VA) approaches can be effective tools for making sense of large datasets and perform complex tasks. Their strengths come from a tight integration of automated analysis methods and visual interactive interfaces [TC05]. In recent years, many VA approaches have been proposed to solve data analysis problems in a wide set of scenarios. However, usually benefits come at a price: Automated analysis methods and visualization techniques need to be configured and meaningful parameters need to be set to obtain high‐quality results. Despite the development of guidelines and the adoption of well‐established design patterns [DFAB04, DAREA*18], using interactive interfaces may present many challenges to analysts.

Given these premises, the research community started to develop approaches and techniques to support data analysts during the analysis process. These are known as guidance [CGM*17]. The main aim of guidance is to ease problematic situations and mitigate issues that might hinder the analyst from achieving results, generating insights, and in the end producing new knowledge. Recent studies show that including guidance in the analysis may be beneficial for the user [CGMT18, MSDK12, HB05, JLJC05, CGM19, CAS*18]. Interest in guidance is quite recent and research has just started scratching the surface of this field. Previous work on guidance, in fact, explores and describes just the characteristics of the guidance process [CGM*17, CAS*18]. Only little research exists describing general procedures to implement guidance in practical scenarios.

In this paper, we provide an initial steppingstone to close the aforementioned gap by reasoning about the process of designing effective guidance in VA. In contrast to previous works, we study the problem from the perspective of designers. We describe a framework comprising a list of steps for guidance designers and a set of qualitative requirements to guide the whole design process.

Guidance is a context‐dependent process. Therefore, it is hardly possible to create an algorithm for guidance design consisting of concrete instructions while being applicable to any analysis scenario. Instead, we provide a framework that points designers to important considerations in the context of guidance design and guides them through this process in a step‐wise manner. We complement the framework with a set of design requirements that should be satisfied to make the design effective and obtain a user‐tailored solution.

To demonstrate the applicability of our general methodology, we describe three design examples and a comprehensive design walk‐through by a VA expert who was not involved in the development of the framework. The first example is about guiding users in the exploration of cyclic patterns in univariate time‐series data. The other two examples are set in the application domains of engine testing and financial fraud detection.

In summary, the value of this work comes not only from the development of a general framework, but also from the discussion of threats, risks and possible countermeasures thereof that could arise during the design. Our contribution is thus three‐fold:

- We provide a general framework comprising a step‐wise procedure for designers aiming at designing *effective* guidance in VA approaches.
- We describe possible countermeasures to risks and threats that could arise during the design process and support an effective implementation of design requirements.
- We demonstrate the value of our framework by applying it to the design of guidance for VA. In this context, we describe challenges and combine them with an appropriate design of guidance solutions, thus showing the applicability of our framework.

## 2 Related Work

This work is mainly focused on guidance in VA [CGM19].

The research of guidance has quite a long story. Its roots are in human–computer interaction and mixed‐initiative visual data analysis [Sil91, Hor99]. Guidance in VA was born from the need to assist and support users during interactive analytical work. It has been defined as *‘a computer‐assisted process that aims to actively resolve a knowledge gap encountered by users during an interactive visual analytics session'* [CGM*17], p. 2]. This definition contains three key aspects: First, guidance is a continuous effort that runs alongside the regular VA activities. Second, guidance addresses a knowledge gap, which captures the discrepancy between what needs to be known to make analytical progress and what is actually known by the user, such as which visual/analytical methods to use, how to set parameters or how to explore and get insights from the data. Third, guidance is not static, but it reacts to a dynamically changing interactive analysis session.

If done properly, guidance can support the VA process in different regards. Guidance can help to inform, mitigate bias, reduce cognitive load and it can be beneficial for training, engagement and verification [CAS*18].

In the past, a number of characteristics of guidance approaches have been identified [CGM*17, CAS*18, SSMT13]. These characteristics primarily cover aspects of why guidance can be provided and how it should be enacted (e.g. the degree of guidance, the input based on which guidance is generated and the way it is communicated). In this regard, the concept of *knowledge gap* acquires the most important role in designing and implementing guidance methods:

In fact, many issues, that is, knowledge gaps, may arise for the user during the whole analysis process. Likewise, multiple kinds of guidance can be envisioned. To support the user in solving such knowledge gaps, the design of guidance certainly includes the choice of an appropriate user interface, but is not limited to it [DFAB04]. It also includes the design of an intelligent, knowledge‐based system which possibly encompasses the creation of a knowledge base and a reasoning mechanism, determining what kind of knowledge should be provided to fill the gap and let the user continue the analysis. The user interface design concerns the way *how* to provide the necessary knowledge to the user, whereas the intelligent system design focuses on *what* and *when* to provide guidance to the user.

There are several specific examples where guidance has been applied successfully to assist users [GST13, LMS*12, MSDK12, SSL*12, SLG*14, CGMT18]. For instance, Kandel *et al*. [KPHH11] designed an approach that guides the user towards the selection of appropriate data transformations based on the type of data under analysis. Gotz and Wen [GW09] developed a behaviour‐driven approach that supports the analyst in selecting the most appropriate visualization for a given analytical task. Bernard *et al*. [BDV*17] provide guidance to the process of labelling human motion data through the use of unsupervised algorithms. Gladisch *et al*. [GST13] supports the exploration of hierarchical graphs by using a flexible degree‐of‐interest function. May *et al*. [MSDK12] guides the user towards interesting regions of large graphs.

Guidance can be done in many ways and for different scopes. Among the possibilities, often user feedback (either implicitly, or explicitly) is considered. Also, guidance can be informed heuristically, based on data and view quality measures. To date, many quality measures have been introduced. For a review, please see [Ber11, BBK*18].

In summary, the literature seems to be in agreement on the potential benefits and the general requirements of guidance. However, there is no unified framework for designing effective guidance in VA. Based on a thorough inspection of existing work [CGM19], our research aims to narrow this gap in the literature by proposing a design framework built around questions that a guidance designer has to address when developing guidance for VA.

## 3 Motivating Example

To further motivate the need of a framework for guidance designers, let us consider an example of analysing multiple time series describing a football (soccer) game [AAB*17]. At first, we describe briefly the scenario and its challenges, focusing on data, users (i.e. analysts) and tasks [MA14]. Then, we change perspective and discuss the example from the point of view of designers by listing factors to be considered when designing guidance.

**Figure 1:**
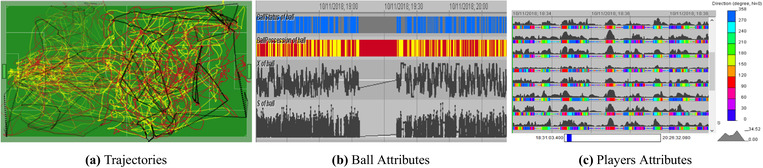
Analysis of soccer matches [AAB*17]. We motivate the need of a proper guidance design: (a) Fragments of trajectories of the players and of the ball for a selected time interval of 5 min length. Finding coordinated movement behaviours by looking at trajectories is complicated due to the overcrowded visualization. (b) Time series representing ball attributes, for instance, ball status (in play or out of play), ball possession (which team), X‐coordinate, and speed. Due to the high frequency of the data, it is difficult to spot patterns at first sights. (c) Time series of attributes of the players. The need to consider multiple attributes makes the discovery of behaviours and patterns complicated.

The dataset under consideration already offers challenges originating from the relations among the multiple variables and the temporal axis. We list various time‐variant attributes derived from the trajectories of the players and the ball (see Figure 1):

- Attributes of the players: speed, movement direction, distances to the two goals, distance to the centre of the own team, etc.
- Attributes of the ball: in play or out of play, which team possesses it, speed, direction, distances to the goals, etc.
- Attributes of the teams: dimensions along and across the pitch, distances from the centre to the goals, mean distance between the team members, mean distance to the nearest opponent player, area of the intersection with the opponent team, etc.

We consider analysts investigating the tactics of the teams in terms of coordination between the movements of the team players. This task consists of detecting correlations between multiple time series of the players‐related attributes. The specific challenge is that different patterns of coordination may be used in different kinds of situations. For example, after a team gains the ball in the central part of the pitch, the adopted tactics may be expansion of the team to the sides of the pitch, but the team may behave quite differently after gaining the ball close to the own goal or close to the opponents’ goal. Hence, the analysis requires extraction of subsets of situations with particular characteristics (regarding the attributes of the ball and the teams) and applying correlation analysis to these subsets.

We assume that analysts have sufficient domain knowledge to select the relevant attributes to investigate specific tactics. However, we also suppose that the analysts may not know the following things.

- Data selection: how to set query conditions to select situations with particular characteristics?
- Methods: what is the class of techniques that can be helpful to detect and analyse coordinated behaviours? What specific methods are suitable and how to set their parameters?
- Interpretation: how to interpret the results of the methods, that is, translate numbers into concepts? How to see and explore the coordinated behaviours corresponding to these results?
- Evaluation and validation: how to assess the coherence of a pattern derived from a group of situations across the individual situations (i.e. how much variance exists)?
- Comparison: how to compare the patterns derived for different groups of situations or for different teams?

Multiple issues might occur during the analysis. From the perspective of designers, these are the possible knowledge gaps that need to be anticipated and addressed when designing the guidance. The benefits of an effective design resides in a positive solution of such gaps and, as a consequence, in an easier time for the analysts using VA tools. Therefore, the main question arises:

How do we design *effective* guidance [CGM19] to support a positive analysis outcome? What questions and what criteria should guide the development of an effective guidance solution?

To provide a satisfactory answer to these questions, we start by making a couple of considerations about the given analysis scenario (see Section 4.1).

The above list suggests that different knowledge gaps may affect the analysis at different moments. Analysts wishing to analyse the data should be aware of such issues and know how to overcome them. If this is not the case, then analysts should at least be able to ask for, or rely on, guidance. Hence, we assume that a first important aspect designers should consider is how to incorporate mechanisms to detect problematic situations and let the analysts ask for guidance. The presence of biases also confirms that guidance needs to be tailored to the needs of specific users.

Sticking to the described scenario, considering the specifics of the data (i.e. multivariate time series) is especially relevant for supporting data selection and for suggesting the necessary methods. An important aspect guidance designers should consider are the issues related to the choice of appropriate parameters for the analytical methods involved. Analysts should be supported in setting conditions for time steps preceding or following a given time step (e.g. ‘team B possessed the ball in the previous time step') and specify the minimal length of the time interval in which a given combination of conditions must hold (e.g. ‘the ball possession duration of team A must be at least 5 seconds'). Guidance may support this process by proposing appropriate parameter settings. However, analysts might not be fully satisfied with them. Hence, a further aspect a guidance designer should consider is the provision of proper means to steer the guidance process in the eventuality that the guidance provided does not satisfy completely the needs of analysts.

Finally, as designers, we could expect the user to be a domain expert, which implies the use of appropriate visual means to visualize and conduct the analysis. For instance, the use of specific means to visualize the field, the trajectories of the ball and the players, without interfering with the analysis dynamics. In this context, another important aspect to address is how to encode the guidance in the analysis process without distracting the user and disrupting the analysis flow.

Analysing this example shows that multiple factors are to be considered when designing guidance. So far, we listed just some of them that should be taken into account when designing guidance for the analysis of time‐varying soccer data. In the following, we formalize, abstract and complement these aspects in a general design framework. At first, we describe a list of requirements that should guide designers when dealing with guidance. We then illustrate a step‐wise procedure to design guidance supporting these requirements.

## 4 A Framework for Guidance Designers

Guidance is described as a closed loop [CGM*18]. On the one hand, the system provides possible solutions to mitigate problems arising during the analysis (i.e. guidance for the analyst). On the other hand, the analyst guides the system by steering the process along the desired analysis path (i.e. guidance for the system). Our framework considers both sides of this process.

##### Methodology

Before introducing the framework, we want to provide a brief overview of the procedure we followed to derive it. The framework was built from multiple sources followed by an iterative refinement process. Initially, we performed a deep investigation of the literature. By performing an analysis of the literature on the topic, we developed an initial understanding of the factors that should be considered when dealing with the process of guiding visual analysis. As a result of this initial phase, we produced a raw list of steps and a description of their interdependencies. We later confronted such initial output with our experience in the field. Having already developed guidance approaches and dealt with similar challenges in the past helped us in refining the initial output into an ordered list of steps and design requirements.

### 4.1 Requirements

In general, our framework is aimed at facilitating the design of *effective* guidance. To support effectiveness, designers should check not only that the guidance is effective for accomplishing the analysis objectives, but that it is also communicated to the analyst at the right moment by appropriate means. Therefore, in the following, we list a set of requirements we think should be met during the design process, to support effectiveness, and make the guidance accepted and user‐tailored. Designers are usually confronted with multiple design alternatives during this process. *We believe that the identified requirements could serve as a guideline to choose among such alternatives*.

We based the requirements on an initial discussion by Ceneda *et al*. [CGM19], who identified a set of characteristics that should concur to the goal of effective guidance. We complemented and further elaborated these characteristics to derive the following design requirements.

R0‐ ***Effective*** guidance is what we see as the end goal of the design process. The designed guidance should be effective for a given task and a given user. To obtain such result, a number of requirements have to be supported:
R1‐ ***Available***: *Guidance is there for you*—Users should be aware that guidance is available and that support can be provided or requested at any time. Designers should make available interactive means to request guidance and appropriate visual means to convey guidance.
R2‐ ***Trustworthy***: *Guidance will help you*—Any generic data analysis task includes a certain degree of variability. Guidance should be regarded as a support to overcome the uncertainty involved and not being a source of further confusion. Designers should take care of specific ways to encode and provide guidance to make it trustworthy and accepted by users, and not an additional source of misinformation. Trust, once lost, is hard to restore.
R3‐ ***Adaptive***: *Guidance will adapt to the situation*—Usually, as the analysis evolves so do the problems users encounter. The guidance system must know what the actual state of the analysis is, in order to deal with dynamically changing knowledge gaps. Designers should implement mechanisms to capture the analysis phase, provide interfaces for inferring the knowledge gap and provide guidance accordingly.
R4‐ ***Controllable***: *Guidance can be tuned if necessary* and the user needs to be in control of the analysis—Guidance is a mixed‐initiative process [Hor99]. Therefore, the designed solution should enable users to steer the analysis, choose between alternative recommendations, turn off the guidance if not needed or provide means to ask for assistance in the first place.
R5‐ ***Non‐disruptive***: *Guidance will not annoy or mislead you*—A final quality that we expect to be supported by the guidance process is that it should not disrupt the analysis flow and the analysts’ mental map. The guidance should be provided without having users to exit their state of flow.

**Figure 2:**
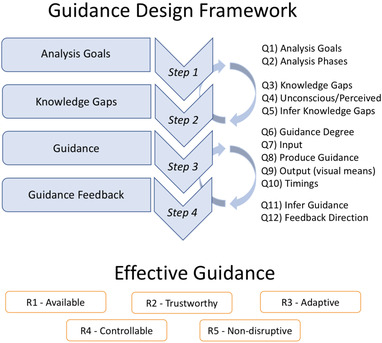
The guidance design framework. The framework aims to support the design of effective guidance (R0, see Section 4.1). We list a set of steps (Step 1–4) as well as quality criteria (R1–R5) that should guide designers during the design process. The arrows going back and forth between the steps illustrate the iterative nature of the design.

In the following, we introduce four major general steps that should be completed in order to come up with an appropriate design of system and user guidance (see Figure 2). To make the framework easier to understand, we complement the description of the individual steps with examples from our introductory soccer scenario and with a description of risks that might arise during the design, as well as possible countermeasures.

### 4.2 Step 1: Analysis goals

When designers approach the problem of providing guidance, they should start with identifying the analysis goal. The following questions should be answered:

*Q1 ‐ What are the analysis goals?*

*Q2 ‐ In which analysis phases issues might occur?*

Different analysis goals may require different guidance solutions. In the case of the soccer match analysis (Section 3), the goal is to identify coordination patterns in the tactical movements of players.

The process of pursuing a given analysis goal can be divided into a sequence of analysis phases that the analyst needs to go through, for example, explore the data, evaluate findings and document results. Therefore, guidance designers should not only consider the analysis end goal, but also examine the different sub‐tasks that the analyst has to deal with in order to reach the end goal. Designers should then implement strategies to infer such phases, which is crucial to design adaptive guidance (R3). Breaking down the analysis process into single atomic tasks will allow the design of guidance for isolated problems and compose them to solve more complex analysis tasks.

According to Andrienko *et al*. [ALA*18], the data analysis process is composed of data preparation, data analysis and model development and evaluation. A further subdivision of the preparation phase includes: (1) understanding the data and (2) pre‐processing the data before the analysis. A subdivision of the analysis phase includes: (3) exploring the data and (4) developing a model. Finally, the data model is (5) evaluated and tested against the work hypotheses. Guidance may be needed at each of these phases, as many issues might arise along them, as shown also by the motivating example (see list of knowledge gaps in Section 3).

#### 4.2.1 Step 1 —Risks, Threats and Countermeasures

Possible risks derived from a non‐satisfactory execution of this design step are **overestimation**, **underestimation** and **misunderstanding** of the analysis goals. This means that the designer may identify too many or too few activities/tasks/goals requiring guidance. Even worse, a wrong design can also be a consequence of human errors, for instance, designers misunderstanding the analysis goals. To mitigate the latter risk, adaptive guidance mechanisms can be devised. For a detailed discussion, see Section 4.3.5. In case of overestimation, the end user (i.e. the analyst) could be bothered by the excess of support, which may lead the analyst to ignore the provided guidance and nullify its benefits, thus going against R1. The threat deriving from underestimation instead is the design of insufficient guidance. The underestimation could lead to a lack of proper support for critical tasks. In general, underestimation and overestimation run against the general aim of designing trustworthy (R2) and non‐disruptive (R5) guidance. In such situations, an effective strategy for supporting a proper design requires close collaboration with domain experts who could provide crucial information about how to structure the tasks and support the identification of analysis objectives. Furthermore, the implementation of means for the user to control (R4) and to fine‐tune the guidance might be a viable alternative solution to counteract such threats.

### 4.3 Step 2: Knowledge gap

After identifying the analysis goal and the different analysis phases, guidance designers need to understand possible knowledge gaps [CGM*17] arising in the completion of those goals.

At this stage, the following question has to be asked:

*Q3 ‐ What knowledge gaps might hinder the analyst from proceeding the analysis?*

A knowledge gap refers to the lack of knowledge or information that makes it difficult to complete the analysis or a certain phase of it, as identified in the previous step.

#### 4.3.1 Structure or execution?

A first general distinction we should make as designers is whether we think the analyst may need help to reach the analysis objective, or to define a sequence of operations to reach it in the first place. In other words, designers should reason whether the knowledge gap is a problem of *structure* or *execution* [Sil91].

In the first case, the knowledge gap relates to finding the correct operators (e.g. algorithms, visualizations) and a combination thereof, in order to obtain the desired results. At this regard, a viable solution may consist of listing the available operators, as well as informing the analyst about alternative options that might serve the same purpose.

In the second case, the knowledge gap is related to the execution of the conceived plan, for instance, a structured sequence of operators, as detailed before. This could include the choice of parameters for each step. The execution of a given sub‐task is related to the decisions taken by the analyst in the previous analysis steps. Therefore, a designer has two alternatives: directly guide the execution of such steps, for instance, guide the choice of proper inputs and support the analysis of the obtained output, or opt for an informative solution: provide the analyst with all the important information about the input as well as details about the possible expected outcomes, and let the analyst take the decision. In the end, it is a matter of giving the analyst more or less freedom.

#### 4.3.2 Types of knowledge gaps

Another way of reasoning about the knowledge gap is considering its type. Four different types of knowledge gaps, which may appear at any phase of the analysis, can be identified [CGM*17].

**1) Data**: the lack of knowledge about the data. This kind of problem generally affects the pre‐processing phases. In our soccer example, we may see the analysts having problems with understanding the relationships between the variables (e.g. the ball position) and the time axis (see Figure 1b). A knowledge gap in the data domain may also affect other analysis phases, for instance, data exploration. In this case, data issues may be related to identifying specific data cases that are helpful to validate hypotheses, for instance, finding data subsets that describe certain known tactics.

**2) Tasks**: the lack of procedural knowledge (i.e. what are the steps) to complete a sub‐task or to reach the analysis goal. For instance, finding sequences of interactions (i.e. selections, filtering) in relation to different features, like ball possession, shots and foul events with the goal of analysing team behaviours.

**3) VA methods/algorithms**: the lack of knowledge about what visual and analytical methods to apply, what algorithms to choose and how to set their parameters. For instance, the analyst might have problems to compose a visual summary of the soccer match, since stacking trajectories may lead to visual occlusion and clutter (see Figure 1a). Methods and algorithms are needed at multiple analysis phases, such as data‐preprocessing or model building (e.g. setting the parameters for clustering the positions of different soccer players). Other issues might occur when selecting appropriate features during the segmentation of the time series, to abstract, for instance, the data into events of the soccer match (see Figure 1b).

**4) Knowledge and insights management**: the lack of skills in interpreting patterns or the organization of the knowledge itself. This knowledge gap is related to merging, interpreting, labelling and managing the findings to generate insights and new knowledge. For instance, translating patterns perceived from the visualization or discovered by algorithms into the domain concepts.

At this stage, we have considered analysis phases and knowledge gaps thereof. Related questions a designer should consider are how to identify knowledge gaps and understand if the analyst perceives them during the analysis. These considerations might in the end lead to completely different guidance solutions.

#### 4.3.3 Perceived and unconscious knowledge gaps

Having identified the knowledge gaps, the next question to be asked is the following:

*Q4 ‐ Are analysts aware or unaware of their knowledge gaps?*

As designers, we should think whether the knowledge gap is perceived by the analyst. A perceived knowledge gap is one that analysts are aware of. The opposite is considered an unconscious knowledge gap. An unconscious knowledge gap related to the *data*, might be, for instance, that the analysts are unaware of missing values, noise, or outliers in the data. This may affect the analysis outcome. There might be unknown biases, and analysts might observe false patterns in the data. Unconscious knowledge gaps might lead to wrong interpretations and conclusions. If the unconscious knowledge gap is related to *tasks*, analysts may use wrong procedures to pursue the analysis. In the case of an unconscious knowledge gap related to *VA methods and algorithms*, the analyst might simply use the wrong parameters or select parameters unsuitable for the task. If the unconscious knowledge gap is related to *knowledge and insights management*, analysts might be confident about specific observations made and conclusions reached when instead the initial hypotheses would need to be re‐evaluated, for example, by looking at the data from another perspective. An unconscious knowledge gap should be treated with special care, since it may reduce the acceptance of the guidance. To prevent this, guidance designers should consider ways to make analysts aware of problematic situations before providing the guidance. Solving issues at this stage is a way to increase trustworthiness (R2) and to achieve non‐disruptive solutions (R5).

#### 4.3.4 Identification of the knowledge gap

The correct identification of the knowledge gaps is related to supporting the design of non‐disruptive guidance (R5). A wrong or incomplete identification may lead to wrong or sub‐optimal guidance that can lead to unexpected analysis outcomes.

*Q5 ‐ How can potential knowledge gaps be identified during the analysis?*

The easiest solution is to let the analysts enter the knowledge gap directly. This solution works well when analysts are aware of the knowledge gap, and know that guidance is available (R1). Conversely, the knowledge gap could be indirectly inferred from the analysts’ actions when working with the visualization by analysing the interaction behaviour. For example, a user that fiddles quite a while with the user controls of a parameter might indicate that the user has difficulties setting a suitable parameter value. To summarize, two main mechanisms can be identified:

- Knowledge gap interface: Enables *conscious* analysts to communicate the knowledge gap to the system. This is useful only if analysts are aware of their knowledge gap.
- Knowledge gap inference: Enables the system to derive the knowledge gap from analysts' behaviour. This is particularly useful when they are not aware of their knowledge gaps.

#### 4.3.5 Step 2 —Risks, threats and countermeasures

Similarly to the previous step, possible risks emerging from a non‐satisfactory execution of this design step are the underestimation and overestimation of the possible knowledge gaps that may arise during the analysis. A viable solution to this problem would be the identification of critical analysis scenarios. These correspond to those moments in the analysis in which it is mandatory for the analyst to take decisions. If the end user is not required to take decisions and to reason about alternatives, guidance is not needed. The identification of these critical moments is crucial to avoid such threats, since these are the situations in which knowledge gaps might occur.

Underestimation and overestimation are related to the **completeness** of the designed guidance solution. This means that a major threat to the design comes from a mismatch between the analysts' needs and the guidance conceived to solve such situations, which conflicts with R3. Although this represents a formidable challenge for research, from the practical point of view, this risk needs to be minimized when designing guidance. While it is hard to guarantee that a guidance solution is complete, in the following, we provide suggestions to minimize this threat:

##### Design for the top‐N knowledge gaps

To improve design completeness, as an initial step, designers could start thinking of guidance to cope with the most problematic knowledge gaps, and thus, design guidance for the majority of crucial cases.

##### Design adaptive guidance

In a second step, designers could aim for *adaptive* guidance mechanisms that could learn as the system is being used [Sil91]. In this way, designers should not worry about incorporating all the pre‐defined content and rules (i.e. what to do when X or Y happens), but just define the boundaries in which the guidance can be provided. Machine learning techniques might be a good choice for such learning mechanisms.

##### Let the analysts guide themselves

The generation of dynamic content cannot always be pursued. A learning system might of course also fail in some situations, which may result in the provision of incomplete, or worse, wrong guidance. Therefore, designers need a backup solution for such cases. To avoid the aforementioned problem and at the same time improve the completeness of the design, we solicit the design of mechanisms to help analysts guide themselves. In practical scenarios, this corresponds to providing analysts with all the necessary information they might need to make a legitimate choice. This could be helpful for instance, during exploratory analysis, when analysis goals cannot be precisely defined. Although this solution puts a large part of the burden on the analyst, we argue that, in case of doubt, it is better than providing the analyst with imprecise recommendations.

### 4.4 Step 3: Guidance generation

This step deals with designing the appropriate guidance needed to narrow or resolve the knowledge gaps (Step 2) and get closer to the analysis goal (Step 1). Designers have to consider the characteristics of the guidance required as well as the moment to provide it.

#### 4.4.1 Guidance characteristics

We structure the guidance characteristics according to the work by Ceneda *et al*. [CGM*17].

##### Guidance degree

Designers should decide how much support the analyst needs. Proper mechanisms are needed to adapt the guidance degree to the current analysis situation. The questions are:

*Q6 ‐ What degree of guidance is needed? What mechanisms can be employed to switch among different degrees?*

The choice of the guidance degree is mainly influenced by the analysts' prior knowledge. Too much or too little guidance might be detrimental, depending on the user knowledge and experience. Consequently, a dynamic degree is preferred, since user knowledge can vary from task to task.

There are three guidance degrees [CGM*17]: orienting, directing and prescribing. *Orienting* guidance provides users with hints so that they can orient themselves and maintain their mental map. It usually makes use of auxiliary means, such as highlighting or transitions between states, enabling users to seamlessly switch the analysis context and pursue different exploration goals. Orienting guidance could, for our soccer example, highlight interesting values or players (e.g. the player who completed the most passes) for further analysis. *Directing* guidance provides more assistance to the analyst than orienting guidance, usually in the form of an ordered list of suggestions. For instance, automatically suggesting and ranking the most prominent events in the soccer game, based on some interestingness measure. Finally, the last degree of guidance aims to *prescribe* a set of actions analysts should take to overcome their knowledge gap. The system could even carry out the actions autonomously.

From a designer's point of view, the provision of the most appropriate guidance degree is mandatory for an effective analysis (R0). Designers should consider all degrees, as well as the design of mechanisms to seamlessly switch between them. The employed guidance degree should match the analysts' knowledge, in order to not be too restrictive or leave too many unknowns. Providing the most appropriate guidance degree (at the right time) is important to meet requirements R2, R3, and R5.

##### Guidance input

Considering the guidance input means considering all the different sources that might be useful to produce guidance. The designer has to ask:

*Q7 ‐ What input is available?*

Usually, different types of inputs are available at the time of designing guidance. The *data* under analysis might be used to extract statistics about the team players. An input that is commonly exploited is a *knowledge* base, for instance, a catalog of labelled soccer events. Another factor that needs to be taken into account is who the end *user* is. The user knowledge—both operational and domain knowledge—has a direct impact on the type of guidance needed. A coach, for instance, might be interested in the tactics of the next opponent. In contrast, fans might seek more information on their favourite club. The *history* of user actions and information about *provenance* may also be useful inputs to generate guidance. Finally, user *preferences* and possible subjective *biases* should be taken into account as well.

##### Algorithms and procedures to calculate guidance

At this point, knowing about the possible *inputs* and the *degree* of guidance, it is important to identify suitable algorithms to compute the guidance output. The following question has to be answered:

*Q8 ‐ What algorithms and procedures are needed to generate guidance?*

Algorithms for producing guidance vary according to the scenarios in which guidance is needed and according to the knowledge gaps. Algorithms for producing guidance refer to *how* guidance is generated and might be different from the algorithms used to identify the knowledge gaps (Step 2).

##### Guidance output

Once produced, the guidance output must be provided to the analyst. Usually we consider visual means, but also acoustic or even haptic output might be helpful.

*Q9 ‐ What are appropriate means to communicate the guidance output?*

In order to support R1 and R5, appropriate means need to be selected. The guidance designer may choose to provide suggestions/hints in the form of simple *text*. Other frequently used expedients to convey guidance are *highlighting and changing colour* of interesting data items [HB05]. *Motion and animation* could also be used to communicate guidance [JLJC05]. However, *glyphs and visual artefacts* are the most common way for encoding guidance suggestions [CGM19].

#### 4.4.2 Identification of the moment to provide guidance

Finally, the last question related to the design of appropriate guidance is identifying the correct moment or time frame to provide it:

*Q10 ‐ When should the guidance be provided?*

One might think that the instants that immediately follow the detection of the knowledge gap are the best option in every situation. However, this may depend, for instance, on the task and on the analysts' behaviour. The choice of the wrong moment to provide guidance may negatively affect the acceptance of guidance (R1) and disrupt the analysis flow (R5).

#### 4.4.3 Step 3 —Risks, threats and countermeasures

Similarly to the previous design steps, we mention threats affecting the design and discuss available countermeasures. Possible risks deriving from a non‐satisfactory execution of this step are: the introduction of **biases**, the choice of a **wrong guidance degree** and the choice of **wrong timing** for providing guidance. A wrong realization of Step 3 would counteract the implementation of trustworthy (R2), adaptive (R3) and non‐disruptive (R5) guidance.

It is well‐known that we, as humans, are affected by cognitive biases [HNM15]. These biases represent a systematic deviation from what is generally recognized as a *rational judgment*. Types of biases can be, for instance, the *confirmation bias* where users tend to stick to hypotheses that comply their way of thinking, or the *repetition bias* in which a user trusts, and thus, remains anchored to repeated procedures. Guidance can help to solve biases but it can also introduce new biases itself. Hence, it is essential for designers to understand users' biases, take them into consideration during the design and think of guidance mechanisms to break systematic, wrong cognitive patterns. On the other hand, it is necessary that the designed guidance does not itself introduce further (unwanted) biases. If we stick to the mentioned example, if a system provides the same guidance suggestions in similar analysis scenarios, then as a consequence the user may learn that in such situations a pre‐defined set of actions can be used to exit a stalled situation. However, if the scenario changes just slightly, the assumption that those actions are still useful may no longer be valid (bias of repetition). Such biases should be recognized by the system and their introduction should be carefully considered by the designers. As a solution, designers could think, for instance, of mechanisms to warn the user about the changed context. Unfortunately, these biases are subjective by nature and a generalized solution cannot be devised for each and every guidance solution. As a general advise, it is recommendable to conduct the design in collaboration with the end‐users. Considering iterative cycles alternating design and evaluation phases could also help mitigate such problems.

Further threats affecting this step derive from an inappropriate provision of guidance, that is, a wrong timing is chosen, and from the selection of an unsuited degree of assistance. It is easy to imagine that providing guidance at the wrong moment may sway the analyst. In the same way, the choice of a wrong guidance degree may frustrate users, limit their actions and nullify the benefits of guidance. Although guidance theoretically might be required at any time, it is worth mentioning that in practical analysis scenarios, when discrete interaction is involved, it is indeed likely that guidance is needed only at distinct points in time. These moments, in fact, correspond to those situations in which the user is required to take a decision or make a judgment [Sil91]. In the absence of these cases, the opportunities to offer guidance are minimal. Therefore, to avoid the aforementioned problems, the role of designers is to identify these decision points in the analysis, to define an order of such moments and define critical decisions. Providing guidance and limit the alternatives available at such points can make a difference in a successful analysis.

In general, we recognize that identifying the precise moment to provide guidance is not always possible. Exploratory analysis is a viable example of this, since its goals as well as the whole process are affected by a great degree of uncertainty. However, also when this is not possible, providing the analysts with orienting guidance, that is, providing all the necessary information about possible actions so that the analyst is able to make an informed decision, can be a suitable baseline solution.

### 4.5 Step 4: Guidance feedback loop

When designers know how to identify the analysts' knowledge gaps and possible guidance solutions to close or narrow them, they need to design means that allow analysts to fine‐tune the provided guidance. Guidance is a mixed‐initiative approach [Hor99], and proper methods to steer the process must be identified. With the aim of designing such feedback mechanisms (i.e. guidance for the system), designers should think of two main aspects: (1) Mechanisms to derive guidance for the system from analysts' actions (usually in the form of feedback). We will refer to this aspect as *guidance inference*. (2) The direction of such guidance: guidance can be directed towards the past or the future. This step is aimed at assuring that the provided guidance is controllable (R4).

#### 4.5.1 Inferring guidance for the system

Interaction is the most common way for analysts to fine‐tune the guidance, for instance, its degree [CGM19].

*Q11 ‐ How can the system derive guidance from the analyst's actions?*

At this design stage, the designer should decide whether sequences of direct actions, or indirect signals, or both should be considered to infer the analysts' feedback about the provided guidance. Two kinds of feedback can be identified:

- Direct feedback: the analyst moves *sliders* or uses other controls for changing the guidance parameters directly.
- Indirect feedback: the analyst acts on the *data*. Analysts move the data, group the data, label the data, which affects the guidance algorithms indirectly.

The literature on using *direct* interaction in visual analysis is vast [AES05, Shn96]. Interaction can be used to provide feedback to the guidance process, too. In other words, the analyst fine‐tunes the guidance parameters by means of interaction with user interface elements, such as widgets and buttons. For instance, if analysts are not satisfied with the data grouping suggested by the guidance system, they may use sliders to adjust the results. The guidance system should hence adapt future guidance results. Usually, single actions are considered. In other cases, the history of actions is contrasted with a knowledge base to extract useful usage patterns [FTIN97].

The second interaction method is what we refer to as *indirect* feedback [EHR*14]. This is the case when analysts do not directly communicate their feedback to fine‐tune the guidance system, but the feedback is derived from their interaction with the data (and not with the widgets). For instance, the analysts' intention to change the data grouping may be indirectly derived from the action of moving specific data points closer to each other, in contrast to the direct use of sliders or widgets. Although direct feedback is the most common method, indirect interaction might open the door for more natural feedback, since it allows a direct contact with the data, which could lead to better user acceptance (R2).

#### 4.5.2 Direction of feedback

In the previous step, we identified the analysts' feedback and ways to infer it. In this step, designers have to identify the direction of the feedback.

*Q12 ‐ What is the direction of the analysts' feedback?*

As mentioned, the guidance directions can be *past* and *future*. Following the literature in cognitive sciences [Dow99], we refer to actions towards the past as *feedback*, and actions taken to call for future guidance as *feedforward* actions. Our idea of feedback, is similar to the one used in cognitive sciences [Dow99], and is related to the concept of relevance feedback. With relevance feedback, relevant items, for instance, the results of a query, are used by the system to provide further guidance to the user. However, in this case, it is the user that guides the system and steers the guidance process. As designers it is important to specify the quality of such evaluation: *positive* and *negative*. Positive and negative feedback are meant to provide a positive or negative evaluation of the guidance the system has provided in the previous analysis loop. Feedforward actions, either positive or negative, should enable analysts to provide hints how they want the guidance to look like in the next guidance loop, and thus, steer and refine the generation of appropriate guidance suggestions.

#### 4.5.3 Step 4 —Risks, threats and countermeasures

A major threat to the design is an unsatisfactory realization of R4: **controllable** guidance, and thus, the provided guidance cannot be controlled by the user. In other words, there is an imbalance between the possibilities offered by the system and the requests of the user: the system guides and forces the choices of the analyst, but the analyst cannot guide and steer the analysis. In some situations, limiting the available alternatives is desirable, for instance, when is recommended to perform a limited set of actions. However, this cannot be assumed as a general design pattern, as the analyst may need a larger set of analytical options and be enabled to deviate from the current line of inquiry. The literature about the science of interaction is vast [DFAB04]. Designers should choose and design the interaction flow considering the analysis requirements and find a suitable balance between restricting and guiding the analyst.

### 4.6 Iterative design of guidance

Having described the different steps and the requirements, we want to make a short digression discussing the iterative nature of our framework. It is common practice in computer science but also in visualization and VA to consider iterative cycles of design, in which a product or a process is cyclically refined with respect to user feedback, in order to obtain a satisfactory result. It is also common that the number of design goals increases or the goals change in the course of this iterative design process. Our framework follows the same strategy by providing the possibility to move back and forth between the steps. For instance, the understanding of the analysis goals might change (Step 1) as guidance mechanisms are defined (Step 3). Our framework proposes a set of qualitative requirements and provides a list of easy‐to‐use design questions for each step of the process. These qualitative requirements and design questions help users to design comprehensive guidance mechanisms even when refining the design multiple times.

## 5 Designing Guidance: Three Scenarios

The framework can be applied to a wide range of scenarios in the context of guidance design for VA.

To make the framework easier to understand for the reader, we illustrate it by describing three examples and a comprehensive design walk‐through. The three design examples are taken from literature. Some of the authors of this paper collaborated to their development in various ways. Instead, the design walk‐through describes a complete design which we performed from scratch using our framework. While the examples should be useful to understand the different aspects considered by the framework, the walk‐through should illustrate a way to instantiate it and make it actionable. Table 1 complements the examples by summarizing the answers to the questions posed in the previous section.

**Table 1:** Questionnaire summarizing the design of guidance in three application scenarios. The questionnaire is based on the guidance design framework. (1) Guidance for cyclical patterns exploration [CGMT18]. The knowledge gap refers to the lack of knowledge regarding the length of the cycles in the univariate time series. The questionnaire shows that while many aspects are considered in this guidance design, question Q12 is not fully answered (n.a.). (2) Condition monitoring and failure detection: The focus is on high dimensional multivariate time‐series data. Guidance is needed to correctly set the parameters of the algorithms to detect anomalies and correlations across events. (3) Fraud detection in financial systems [LGM19]. Guidance is needed to support the analyst in analysing a financial transaction graph and discerning whether such transactions are frauds or regular money movements. The knowledge gap refers to finding parameters and form non‐empty and meaningful queries to the system. Also in this example, the designed guidance is quite comprehensive, as all the questions are answered

	**Questions**	**Cyclical patterns**	**Condition monitoring**	**Fraud detection**
Q1	What are the analysis goals?	Explore cyclical patterns	Anomaly and failure detection	Frauds exploration and confirmation
Q2	In which analysis phases might issues occur?	Exploration	Exploration	Model building
Q3	What is the knowledge gap?	Parameters	Data/VA methods	Parameters
Q4	Is the knowledge gap perceived/unconscious?	Perceived	Perceived, but bias may occur	Perceived
Q5	How can knowledge gaps be identified?	Case study interviews	Case study interviews	Case study interviews
Q6	What guidance degree is needed?	Orienting	Orienting/directing	Directing/prescribing
Q7	What input is available?	Data	data/thresholds	Data/domain knowledge
Q8	Algorithms to produce guidance?	Cycle detection algorithms	Correlation/classification algorithms	Neighbourhood exploration
Q9	Appropriate means to encode guidance?	Glyphs in sliders	Overview/marks	Forbid certain queries
Q10	When should guidance be provided?	Throughout	Throughout	Throughout
Q11	How can guidance for the system be derived?	Direct feedback	Direct feedback	Direct feedback
Q12	What is the direction of the feedback?	n.a.	Forward and backwards	Forward

### 5.1 Exploration of cyclical patterns in time series

In our first example, we address the visual analysis and exploration of cyclical patterns in univariate time series [CGMT18]. For unknown data, it is typically not clear beforehand if and where cycles and patterns exist in the data. This leads to time‐consuming phases of trial‐and‐error searching, where analysts have to spot a possible pattern and then verify its existence in the whole dataset. A purely algorithmic solution to find cyclic patterns is not feasible either. Algorithms to automatically detect cycles are difficult to select and configure. Thus, guidance is designed to mitigate these problem by reducing time‐consuming tasks.

**Figure 3:**
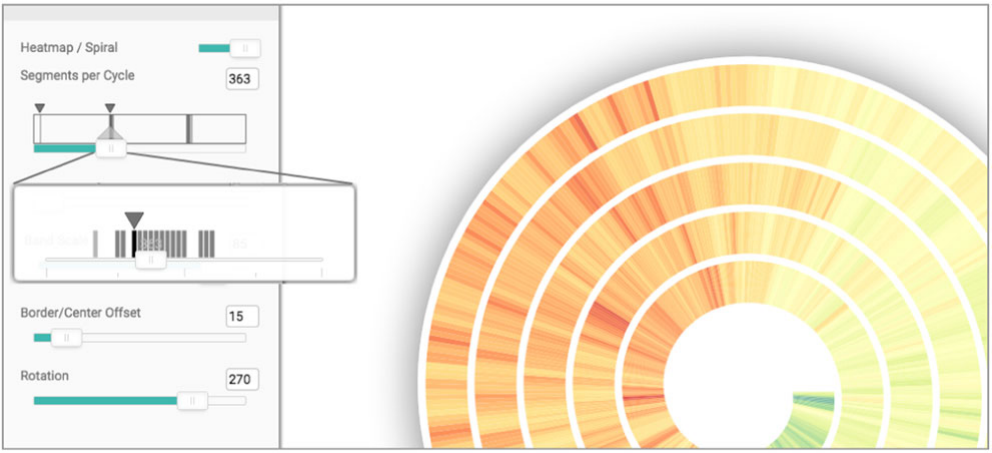
Guidance for exploring cyclical patterns [CGMT18]. Analysts are supported in finding cycles. Suitable cycle length values are encoded in the sliders (see the gray bars on the left‐hand side of the image) that control the visualization of patterns. By choosing the suggested values, cycles appear in the visualization.

The idea is to support the detection of cycles by indicating possible instances of cyclical patterns. This information will guide the user towards configurations that will potentially make cycles visible in the visualization (see Figure 3).

##### Step 1: Analysis goal

This design example is limited to a specific, yet important, research challenge, that is, identifying patterns in cyclical data. We specify this design problem as a sequence of two sub‐problems: The first problem concerns the **exploration** of the dataset. We want to support users in finding cycles and recurrent patterns. The second problem is that, once analysts have explored the data, they have to **build a model** (for instance, understand the regularity of the discovered cycles) and formulate appropriate hypotheses. In this scenario, we imagine that issues might appear in such analysis phases.

##### Step 2: Knowledge gap

We can imagine the analysts being experts in the field. This means that they possess sufficient domain knowledge to interpret the data correctly. However, patterns and cycles might not be known in advance. Hence, the knowledge gap can be framed as a execution problem. Analysts do not know in advance what **parameters** will make such cycles appear. In the design of the guidance solution, methods to infer the knowledge gap during the analysis are not mentioned. Since the design of guidance is limited to a specific research problem, the risks of over/underestimation of the possible knowledge gaps do not exist, in this case.

##### Step 3: Guidance generation

In order to provide an answer to the aforementioned knowledge gap and support a successful data exploration, the guidance provides suggestions of possible parameter settings that would make cyclical patterns visible. Thanks to such suggestions, analysts can configure the visualization in a way that lets them explore the most promising cyclical patterns.

*Guidance degree*. Analysts are supported with **orienting** guidance. The choice of this guidance degree is due to the fact that the analyst needs to perform an exploration analysis. Since the importance of the detected patterns is not known in advance, it is a better option not to guide the analysts directly by providing recommendations (i.e. directing guidance), but rather enable them to make an informed decision. The designed guidance shows the automatically detected patterns but does not enforce any order and provides the analyst with statistics about these patterns. Hence, in this scenario, analysts were allowed to formulate and test their own hypotheses, without being influenced by the provided guidance.

*Guidance input*. The only input needed for the guidance process is the *data* itself. Together with the aforementioned algorithms, the data are used to calculate a list of possible parameters settings that can make the cycles appear, if present.

*Algorithms and VA methods*. In order to produce the suggestions, the guidance process exploits two algorithms [i.e. the chi‐square periodogram (CSP) and the discrete fourier transform (DFT)], which are commonly used for finding cyclical patterns in time series. The DFT provides precise indications of patterns, while the CSP complements them with a probability, which constitutes a source of guidance for the analyst. During the design, we chose these algorithms because they complement each other. The algorithms produce a list of long and short patterns, giving the analyst a nice overview of the data.

*Guidance output*. Once the suggestions are computed they must be visualized. In this scenario, the suggestions are encoded directly using the sliders to modify the visual appearance of the visualization. This choice was made to avoid distracting users from the exploration activities (supporting R5). Furthermore, this choice also reduced the risk of introducing biases, since the suggestions are integrated in the normal analysis workflow. The idea is to assign and visualize the output of the algorithms to the place where analysts have the opportunity to identify them. This implicitly makes also the guidance solution immediately available (R1).

*Guidance timing*. In this specific example, we did not consider time frames to provide guidance, since there are no critical judgment moments. Analysts are required only to judge the different alternatives provided by the system and formulate hypotheses. Therefore, providing the analysts with a detailed list of patterns at the beginning of the analysis was considered a sufficient source of guidance. In a more complex design scenario, we could imagine the system supporting also the choice among alternatives with a higher degree of guidance (e.g. directing guidance). In this case, critical moments to provide guidance could be the moments preceding the choice of a specific pattern, after the analyst has already filtered out the less promising patterns. At those points, directing guidance and recommendations could be effectively provided.

##### Step 4: Guidance feedback loop

In the current iteration, the described guidance solution does not allow fine‐tuning, meaning that it does not allow the algorithms or their parameters to be changed. However, direct feedback could help users to decide how many, as well as what kind of patterns are suggested. This kind of solution, would constitute a *feedback* to the system and could also work to evaluate which algorithm may provide the better results.

### 5.2 Condition monitoring and failure case detection in engine testing

In automotive engineering, the analysis of test data obtained from an engine in a test‐bed is a common task. Engine testing is a key phase in engine development, and serves as verification and validation of engine designs. Typically, engines go through repeated, programmatic test cycles in the test‐bed. For analysis purposes, numerous sensors are equipped, which record characteristic properties of the engine over time. Typically, multiple timelines are used to represent sensor measurements. The primary goal of engineers and analysts is the detection of anomalies as well as their root cause, which may be related to design errors. In this scenario [SMF*20], guidance could be used to reduce the burden on the user to detect anomalies. However, since anomalies may vary, also the knowledge of the analyst is of great importance to detect relevant abnormal events. Hence, a proper balance needs to be found when designing guidance, between user freedom and system restrictiveness.

##### Step 1: Analysis goal

Based on design study interviews, two main goals have been identified for this use case (see Table 1). Under the **exploration** goal, analysts want to test if the engine behaves as expected or is affected by anomalies. Also, identification of correlated and uncorrelated measurement data is important. Depending on the engine design, measurements may influence each other, or be independent from each other. **Model building** involves finding a description for regular and anomalous test states, eventually rules to install for an automatic monitoring. Hence, analysis phases include verification and falsification during exploration and monitoring. A guidance system should be available (*R1*) and adaptive (R3) to these tasks. As the tasks include both data analysis for model building and monitoring for failure cases, the guidance may necessarily be disruptive at times (*R5*) but should exercise the disruption only when needed.

Since engine certification tests are standardized routines, the risk of underestimating (see Section 4.2) the analysis goals almost does not exist. The analysis of test cycles can be easily identified as an exploration task, with the aim of identifying anomalies. As for model building, this goal was introduced in the attempt to partially automate and ease the detection of anomalies, reducing the burden of the analysis. However, a fully automatic monitoring is not possible due to the changing conditions of each test.

##### Step 2: Knowledge gap

Analysts are trained automotive and mechanical engineers. They possess domain knowledge on expected engine characteristics under varying loads and effects of wear over time during the set of test cycles. A first knowledge gap can be framed as a **data** problem. Patterns in the data may represent normal and abnormal engine states. Some are known from experience and training, but for newly developed engines, new patterns may occur during verification and validation. Also, normal and abnormal states can be described not only by single variables, but by combinations of variables and their interplay. There can be abrupt but also smooth transitions between normal and abnormal conditions. This is a large search space. In addition, it may occur that sensor readings become imprecise or erroneous due to failing sensors, which may not immediately be apparent. A second knowledge gap is represented by the choice of the *algorithms* and VA methods. Not all the statistical algorithms are suited to detect a given anomaly. Therefore, analysts should be also guided to choose among alternative detection algorithms. Experts in general are **aware** of the knowledge gap, but may be biased to look for expected variables and at the expense of new variables or their combinations. Trustworthiness of the guidance (*R2*) will be especially important if unknown or unexpected parameters are suggested for analysis. In this step, threats to the completeness of the designed solution could be avoided with the implementation of learning mechanisms for guidance. Well‐known patterns must be taken into consideration. However, a simple rule‐based guidance is not enough and fully automatic analysis is not possible either, due to the changing conditions of each test. The nature of the task asks for the introduction of learning mechanisms, to adapt to new anomaly patterns, in which new rules are dynamically added to complement the existing knowledge base.

##### Step 3: Guidance generation

A key task is to learn what normal and abnormal conditions are. This can be supported by guidance approaches based on showing a suitable degree of similarity between engine measurements over time. Assuming that most of the time, the engine test is in a normal state, abnormal states could show large differences when compared to reference data. This should be done for large amounts of data recordings and many variables. Because data are large, the idea is to support the analysis goals by adding a further level of abstraction on top of the analysis workflow. Instead of analysing hundreds of timelines, analysts will be provided with glyphs that would point to possible problematic situations, reducing in this way the search space.

*Guidance degree*. Two main guidance degrees apply in this use case. The exploration task can be supported with **orienting** guidance. The guidance system continuously evaluates the measurement data for abnormal behaviours and reports occurrences to the users. The approach is designed to point the user to adjusting the thresholds if needed, and hence is controllable (*R4*). Orienting guidance was chosen since a ground truth does not exist for this task. The variable nature of the anomalies makes the task hard to solve in an automated way. Hence, higher degrees of support are not suitable. Providing recommendations (as in directing guidance) is in certain cases not possible and even detrimental.

**Directing** guidance is instead desired for model‐building activities. In this, the guidance could learn from user feedback and subsequently help analysts in choosing the most appropriate algorithms and statistical methods for a given scenario and test cycle.

*Guidance input*. This will be the **data** to be monitored. An initial set of **parameters** need to be set, specifying, for example, thresholds and intervals for the initial anomaly detection. We can assume that rules exist from engineering knowledge and best practices, but they will need to be adapted during the long‐lasting test runs.

*Algorithms and VA methods*. As the dataset is large, the designed solution requires the application of data reduction techniques. This can imply reducing the frequency of the data, for example, by sampling time steps. Also, feature selection methods must be applied to reduce the number of variables. Still, the amount of data may be large. For this reason, in a first analysis step, a measure of the anomalies is calculated [ZKT*17, CBK09]. Analysts could use this measure as a first indication about the presence of possible anomalies in the data.

The designed solution also requires that analytical methods are applied to compare current data with historic data and report larger differences as possible anomalies.

**Figure 4:**
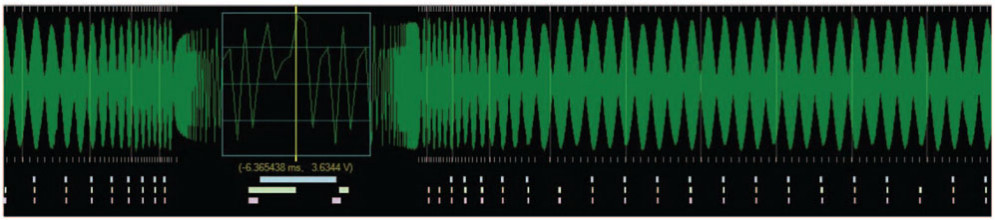
The SignalLens approach indicates detected time series anomalies by level‐of‐detail and markers [Kin10].

To support this task, a regression model is used [LW*02] and the detected features are visualized by level of detail and markers (see Figure 4). However, these algorithms represent just an initial step into the analysis. Since anomalies vary, the system allows for an easy interchange of the algorithms to use in a give scenario.

*Guidance output*. Various visual methods can be used. The analysts, in a normal workflow, are used to simple time‐series visualizations of the recorded sensor measurements. Due to the data dimensionality, the designed solution introduces visual glyphs to give the analysts an overview of possible detected anomalies.

**Figure 5:**
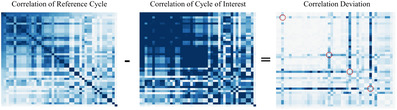
Detecting anomalies by comparing sensor data correlation matrices of a reference test cycle (known normal) with an unknown test cycle. Larger differences in columns or rows hint at anomalous values, in comparison to the reference cycle. By means of visual pattern‐driven exploration of cross patterns deviating sensors can be identified. Four examples of such cross patterns are highlighted in the figure by red circles at the cross's intersection on the diagonal element.

In the following phase, when measurements have to be compared among each other, a scalable approach is based on the visualization of difference matrices to compare the linear relations between sensors of a known normal cycle with those of an unknown cycle. Large differences in the correlation of certain variables hint to possibly anomalous parameters [ZKT*17] and are visible as prominent rows and columns (see Figure 5). A possible risk is represented by the introduction of biases in the analysis: the additional step introduced to help the detection of anomalies should not put additional burden on the user. Hence, to reduce even more such burden on the user, only parameters that are relevant for the current analysis are visualized. Furthermore, guidance has been designed to be included in the normal workflow of the engineers, so as not to disrupt (R5) the reasoning process, but providing support to it.

*Guidance timing*. Timing is relevant. According to the analysis workflow of the engineers, it is possible to identify two main moments when guidance has to be provided. If the engine stops working during the tests, analysts need to be guided to the root cause of the malfunction. The second moment is at the end of the test cycle, when data are analysed and anomalies have to be identified. The two scenarios require similar actions: Analysts should be guided to compare the detected anomaly with the reference model of the engine function and detect design issues.

##### Step 4: Guidance feedback loop

Feedback modalities have been selected to make the analysis controllable (*R4*). User **feedback** on thresholds can help to refine anomaly detection. In particular, not all the detected anomalies are relevant. When the guidance reports such an event, the analyst can fine tune the anomaly detection algorithm, by excluding irrelevant sensor measurements, steering the analysis. Refinement should be possible by the user continuously. Since the analysis is in real time, the knowledge may change anytime but needs to be reflected immediately by the system. **Direct** input is commonly used in this scenario.

### 5.3 Visual detection of frauds in financial systems

Financial institutions are interested in ensuring that illicit operations, that is, fraudulent money transactions, are detected and prosecuted in short time. Fraudulent schemes have nowadays a huge impact on the financial system, impacting the economy and the trustworthiness of the institutions [KCA*16, LGM*18]. To tackle such incidents, financial institutions analyse on average millions of transactions (money movements) per year, the majority of which are legitimate, to detect possibly unlawful schemes and behaviours. The amount of data being analysed does not permit for manual exploration of all cases, and a first labelling of the data is made by automatic algorithms. Afterwards, financial fraud analysts are in charge of making the final decisions, that is, should a customer be accused of fraud? by analysing a subset of transactions. Fraudulent schemes are, usually, complex: This requires to analyse particular structures (patterns) in the transaction graph. The cost and the implications of possible false positives, that is, accusing an innocent person, are high. The usual analysis process, hence, involves a computational system searching for candidate patterns (i.e. possible frauds) and a data analyst who is responsible for analysing the critical cases and deciding final verdicts.

##### Step 1: Analysis goal

Working in close collaboration with financial fraud analysts, a number of tasks and goals was defined. The focus of the guidance in this example scenario is to support specialized analysts in the detection and analysis of possibly fraudulent money movements and understand if they comprise criminal actions. In particular, given a specific bank account, analysts want to understand the flow of money in a quick and effective way. It should be also possible to perform the same tasks considering multiple accounts and their relations, which in this case corresponds to understanding how money is moved through a network of selected accounts. The second task consists of understanding the structure of a transactions network, that is, to understand if the considered transactions constitute fraud or not. To do so, analysts possess the required domain knowledge to judge individual cases. However, they still need support to detect possibly hidden patterns.

The first task is an **exploration task**, that is, exploring the transaction network (compare Section 4.2). The second task is about **building and evaluating a model**, that is, understanding if the given network represents a fraudulent scheme (see also Table 1).

##### Step 2: Knowledge gap

The analysts are bank employees who are experienced in the domain and, therefore, know the data and tasks very well. Analysts can form and send queries to an internal scoring system developed by the bank, looking for suspicious patterns, but they do not know if such queries are meaningful and represent an actual pattern in the transaction graph. This results in long and often unsuccessful trial‐and‐error analyses in which the analyst has to build and refine the queries in multiple stages. In other words, when performing analytical tasks, the analysts' knowledge gap relates to finding meaningful *parameters* and combinations thereof, which will not yield empty or contextually irrelevant queries' results, so to foster an effective *exploration* of the transaction network and detect financial frauds. The designed guidance addresses such issues in that it supports the analyst in forming meaningful and non‐empty queries. The identification of possible knowledge gaps and analysis goals was pursued in collaboration with financial fraud analysts in terms of design study interviews, to minimize the risk of underestimating possible knowledge gaps and to have a clear view of the goals of the project. Designers were able to frame all the possible transaction schemes to a finite set of basic cases. Using such cases as building blocks, analysts are able to construct complex queries without limitations to the query expressiveness and fraud detection capabilities. On top of this, the guidance was designed to avoid the formulation of queries outside these building blocks or cases which are not present in the data.

##### Step 3: Guidance generation

*Guidance degree*—The guidance degree needed in the context of this scenario is a combination of **directing** and **prescribing**. As analysts often face the problem of finding meaningful patterns and formulating appropriate non‐empty queries' results, the designed guidance **indicates** parameter combinations that produce contextually relevant queries and **prohibits** the formulation of queries that lead to an empty result.

*Guidance input*. The first input to the guidance mechanism is **money transaction network data**. Accounts/customers are represented as nodes, while the flow of incoming/outgoing money is represented by edges between nodes. In a first step, an automatic fraud detection system flags suspicious transactions. The analyst is then responsible for delving into these specific cases and confirm or reject their criminal intent. A further input is the **domain knowledge**, which is used to formulate queries, which are meaningful and non‐empty to avoid irrelevant results.

*Algorithms and VA methods*. The manual analysis of the transaction graph is not feasible, as these graphs contain hundreds of thousands of nodes (the accounts) and millions of edges (the transactions). After suspicious transactions have been identified by the automatic fraud detection system (provided by the bank, but we are not allowed to describe the algorithm due to bank regulations), the exploration of the subset of suspicious accounts and transactions is supported by a VA solution. When the analysts form queries to explore the transaction graph, algorithms are used to conduct a preemptive exploration of the neighbourhood of the user‐selected nodes. This exploration allows our VA solution to detect meaningful transaction patterns and consequently support appropriate query formulations. As the analysis makes progresses, the network's neighbourhood is continuously updated and only relevant actions are allowed.

*Guidance output*. The provided guidance approach supports the exploration of the whole transaction network by restricting the parameter space and allowing only for the formulation of meaningful queries resulting in non‐empty output. A prescribed set of queries (also called building blocks) is hard‐coded in the VA solution. These restrictions do not hinder a comprehensive analysis, as they allow analysts to cover all cases present in the data, and thus, to effectively solve their tasks. Therefore, the risk of missing possible frauds is avoided (which supports R3). While fraud detection is always affected by some degree of uncertainty, analysts are trained and aware of it. Hence, the risk of misinterpreting the recommendations is considered low.

**Figure 6:**
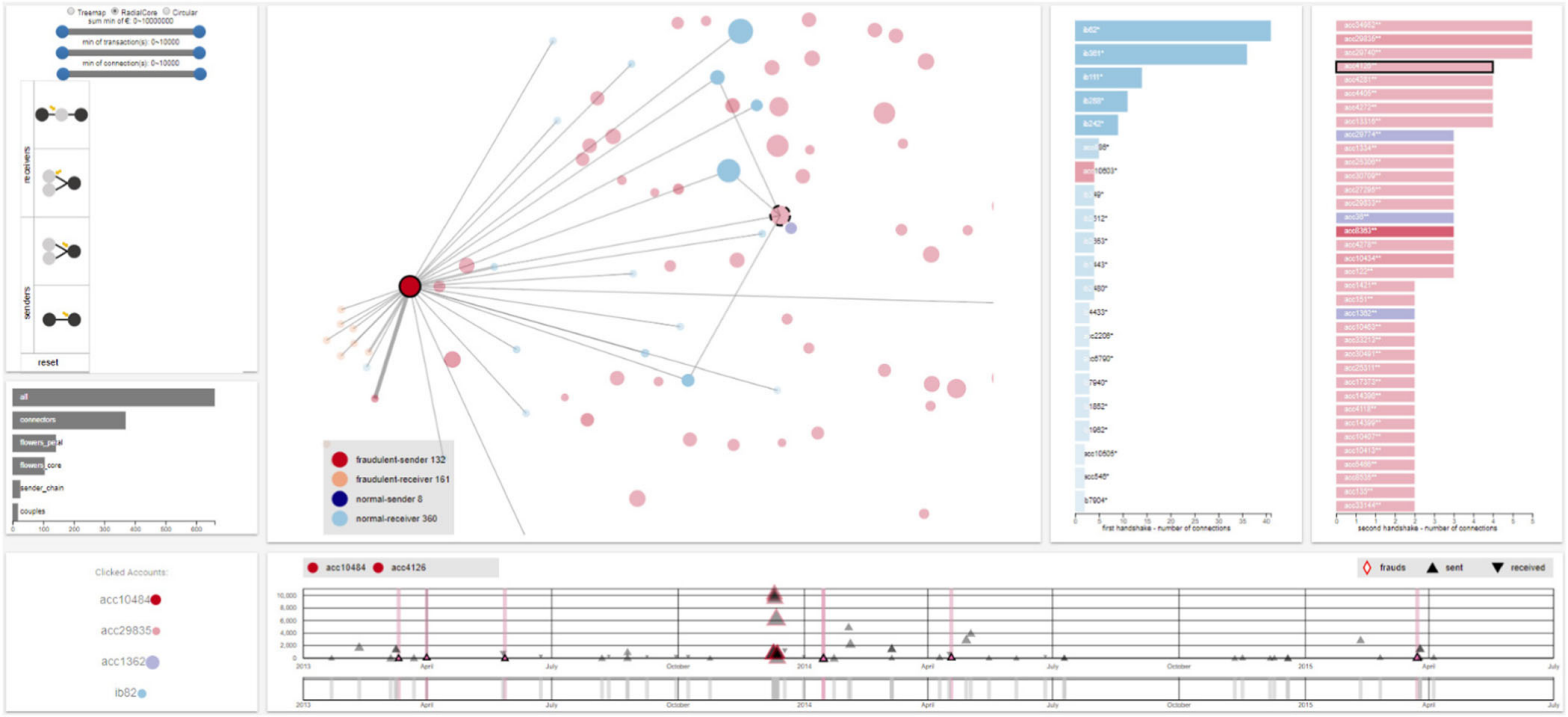
Overview visualization for finding frauds in a money transactions network [LGM19]. The analytical exploration of the network data is supported by allowing analysts to formulate only semantically relevant queries and make the analysis effective. The money transaction graph is displayed in the middle. The left‐hand side of the interface is where the guidance takes place. It shows the building blocks, that is, pre‐defined query components, that the analyst can use to formulate queries. As the exploration proceeds, the list of building blocks is updated, some of them are removed, so that just meaningful and non‐empty queries can be formed.

Fraud analysts are used to working with visualizations, however, not as their primary means of investigation. Thus, an expressive visual encoding for comprehensive visual analysis was designed: The transaction graph is represented as a node‐link diagram in the centre of the visualization (see Figure 6). Another constraint was the ease of use, so to put no additional burden on the analysts (R5). Thus, the guidance suggestions, that is, the allowed operations in a specific time frame were encoded as draggable building blocks to allow formulating queries in a visual way. Whenever a selector would lead to an irrelevant result, it would be grayed out and made unusable. This makes the guidance suggestions immediately available and visible (R1).

*Guidance timing*. When the analyst selects a bank account for exploration, the guidance mechanism explores preemptively network's neighbourhood searching for known patterns. As the exploration proceeds and new nodes are selected, the guidance mechanism updates and provides a new set of meaningful actions. In this sense, the guidance mechanism anticipates possible future actions of the analyst.

##### Step 4: Guidance feedback loop

As mentioned, when analysts select a certain node for exploration, the guidance mechanism automatically explores the network's neighbourhood for detecting potentially interesting money movements. In this sense, the analyst's actions, that is, the selection of nodes to explore, influence the way the system expands the neighbourhood graph. This can be considered as a direct feedback to the guidance. However, the number and type of patterns the VA solution is able to find cannot be directly modified or fine tuned. To overcome this issue, in a current update of the guidance mechanisms, in addition to predefined queries, analysts are allowed to specify user‐defined patterns, providing a finer grained feedback to the guidance mechanism.

Having described three design examples, we can start drawing some conclusion: As shown in Table 1, not all the questions are covered by the examples we describe. The guidance feedback loop is often overlooked or considered just for minor parts of the guidance mechanism. Moreover, the examples we describe offer guidance for selected tasks of the analysis process only. Thus, our examples demonstrate practical solutions to specific issues, rather than guidance designs for comprehensive systems. This is a common issue in the literature: Existing works do not provide any comprehensive guidance solution or complete design examples [CGM19]. In the following, to overcome such issue and to ease the application of our framework to other contexts, we describe a comprehensive and complete step‐by‐step design process.

### 5.4 Design walk‐through: Guidance‐enriched blind source separation

In the following, we describe a design walk‐through which should provide additional means to designers aiming at using our framework. The actual design walk‐through was carried out by a designer, who is an expert in VA but was not involved in defining the framework. After an introduction to the framework, we asked him to perform a complete design. We describe how the designer iterated over all the design steps considering risks, countermeasures and design requirements.

*Problem description*. We ground the design walk‐through in the field of statistics. It happens often nowadays, that a variety of measurements are collected at different locations and times. The reader can imagine various sensors collecting, for instance, temperature measurements, or the fluctuating price of a given good on the stock market, in different regions of the world. These datasets are collections of multivariate time series. Statistically speaking, one of the problems arising when dealing with such data is that it is hard to separate the actual measurement from other signals composing the time‐series. In general, we can think of these other measurements as noise, but a wide variety of signals can add up to form the final value. This is a topic of interest in many domains. For example, physicians need to analyse and detect possible anomalies in an electric cardiogram (ECG) of a pregnant woman in which the heartbeats of the mother and baby are mixed together. This problem can be generally formulated as *separating a signal into its components without any assumption about the characteristics of the original signal*, and it can be shortly called a blind source separation problem (BSS). The task of the designer is to provide guidance and support to solving this problem.

**Step 1: Analysis goal**. The designer bootstrapped the design and approached Step 1 by performing a thorough literature research gaining confidence with the topic. At the same time, the designer had several discussions with the statisticians involved in BSS, which supported the understanding of the BSS problem and analysis goals of the statisticians. In this phase, he reiterated over Q1 (see Section 4) several times and gave an initial answer to Q2 too. Thanks to the interviews, he learnt that usually statisticians would tackle the problem by exploiting mainly the functionalities and packages of R [Tea14] for statistical analysis. By using this iterative method he was also able to keep low the risks associated with this first step (see Section 4.2.1), settling in the end with a compromise between over‐ and underestimation of the analysis goals.

A typical BSS task is usually approached as follows: By using R and other statistical tools, the data, that is, the collection of multivariate signals, are analysed by means of algorithms appositely created to the scope. The output of such algorithms are a set of signals representing the components of the original measurements. In a subsequent step, still through R, the statisticians produce a static visual representation of the results for inspection: They analyse statistics of the output signals and visually inspect them to understand if the result is sufficiently precise and if any interesting pattern is present. If that is the case, the analysis can be considered concluded. However, what often happens is that the analysis of the data has to be repeated multiple times varying the parameters of the BSS algorithms each run, confronting the results with the ones obtained in the previous iterations, and finally interpreting these results to understand if they make sense. Statisticians are usually not interested in finding the optimal solution, which would be anyhow unfeasible to calculate due to the large parameter space, but in calculating one solution that represents a very good, yet not perfect, estimation of the original signal. This statisticians' workflow resembles typical VA tasks [SSS*14]. However, very little emphasis is given to use visualizations or a comprehensive VA methodology. They use static images, which do not provide an easy overview and comparison. Hence, the designer decided, in line with R5, to give further emphasis to the visual means and support of the analysis, which were already a part of the workflow, but enhancing it by using a VA methodology and including guidance in the loop.

**Step 2: Knowledge gap**. While understanding the BSS problem and the statisticians' workflow, the designer moved towards the fulfillment of Step 2. Hence, in parallel to questions regarding the single analysis phases, the statisticians were also interviewed about possible problems they might encounter while completing their tasks. This led to defining a list of possible knowledge gaps, as required by Q3. In total, the statisticians mentioned three main knowledge gaps (defined as KG‐1, KG‐2 and KG‐3 below). All of them can be framed as problems of execution. These will be addressed by the guidance. The analysis workflow is instead already very well structured and defined.

Statisticians have to modify several times the parameters of the BSS algorithms in order to complete the task. Although they can be considered as experts in that they know exactly the meaning and the influence of different parameter combinations, still the *parameter space* is huge (KG‐1), which hinders the possibility of an exhaustive search. The second and third knowledge gaps relate to the *exploration* (KG‐2) and *interpretation* (KG‐3) of the results produced in the first phase. As the algorithms compute the components of a given signal, the results have to be compared with previously obtained components, and their statistical properties have to be compared among each other to judge the goodness of the new obtained output. This requires to maintain and/or remember a collection of previously computed results, which in the original workflow required long back‐and‐forth exploration of statistics and static visualizations in R. Additionally, issues arise when analysts have to consider the computed results in the light of a specific context. Analyst must, in fact, possess not only knowledge about statistics, but also about the data domain. Similar results might be considered useful or totally useless according to the domain of the data. These domain competences concur to determine if a computed signal is a good representative of the original one. The same consideration applies also to the choice of parameters. For instance, some parameter combinations might make more sense than others according to different domains.

After some meetings, the designer had the impression that the statisticians were pretty aware of the knowledge gaps (Q4). In this regard, in earlier iterations of Step 1 and 2, the designer discovered that some of them often relied on some sort of rule‐of‐thumb methodology to solve the tasks. Therefore, from that point on, the designer decided to proceed in this promising direction, and shed more light on such established but implicit practices to see if it was possible to formalize and exploit them, solving KG‐1 and KG‐3.

**Step 3: Guidance generation**. As the designer gained some insights regarding possible knowledge gaps affecting the analysis, the designer looked into possible solutions to solve them. Step 3, which aims at designing guidance for the detected knowledge gaps, was initialized by analysing the types of input available as well as the types of guidance that could be produced. Three inputs are mainly available to the guidance process (Q7): the data, the implicit knowledge of the statisticians, and the domain knowledge, depending on the application scenario. The data can be directly exploited: statistics can be extracted and used to give the statisticians an early idea of which parameters to choose or how to interpret the results obtained. The domain and the statisticians' implicit knowledge, as the word suggests, is not readily available. Hence, the designer looked into ways to formalize and make this knowledge explicit so that it could be used to support the exploration and the interpretation of results. In particular, this will come useful to support KG‐1: the guidance will suggest possible parameters based on the data domain.

The visual analysis tool was designed to easily integrate the design framework into the normal design flow of any visualization environment. Its step‐wise structure allows designers to integrate the two processes. Hence, the designer sketched the visual appearance of the tool to accommodate both the guidance and the user interface widgets. A multiple coordinated views approach was chosen, which supports the two main perspectives: (1) data selection and parameters imputation which should address KG‐1 and partly KG‐2, and (2) the results visualization which should allow easy exploration (KG‐2) and interpretation (KG‐3) of the results. The designer had a further interview round to gain a deeper understanding of the tasks. A new iteration over Step 1 was carried out and a list of the operations necessary to solve the tasks was defined. In total, two critical operations were identified: We describe them as well as the guidance designed to support them, next.

The issues for the statisticians start at an early point of the analysis, since they are immediately called to determine the granularity of the data and the input lags separating the input signals. However, they know nothing about the data in such early phases, which usually reduces the analysis to long iterations of random parameters selections and results inspections. To help them, the designer thought of guidance which can be framed as **orienting** support exploiting the data input (Q6–Q7). As the user loads the data, the tool automatically calculates statistics about the loaded signals and immediately visualizes them to inform the parameter selection. The auto‐correlation of the different input signals can be, for instance, displayed as a line chart (Q8) to determine a proper lag value that separates the input measurements and an appropriate granularity. Hence, following the statisticians' workflow, the tool was designed to calculate automatically such statistics and arrange them in the visualization to facilitate their work.

**Figure 7:**
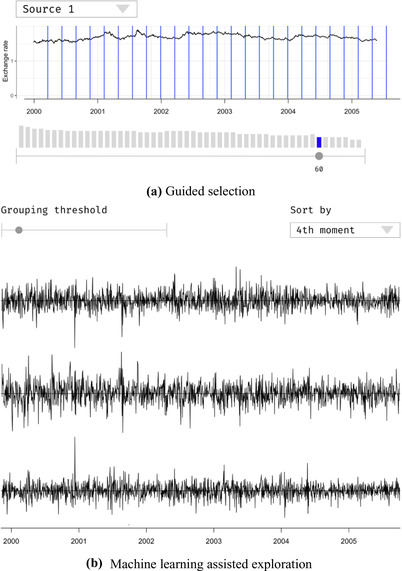
Guidance‐enhanced blind source separation (BSS). We enhance with guidance the task of separating a signal into its basic components. (a) The area above the sliders used to input parameters is used for showing the impact of different parameter choices to users, hence informing them about the possible outcome of a parameter selection. (b) Thanks to classification algorithms the output of the BSS is classified to enhance the exploration and interpretation of the results.

In the current design iteration, the guidance hints were integrated directly in the sliders used to select the parameters of the BSS algorithm (see Figure 7a), in a way that does not distract the user and makes the guidance readily available (R1–R5). A small area above the sliders is reserved to visualize such hints (Q8). The guidance embedded in the sliders acts by showing to the user characteristics of the parameter space. In addition, the analyst can easily modify what statistics should be considered for the guidance, enforcing R4. The guidance degree can be framed as orienting support (Q6).

The designer considered an additional, more advanced, solution for supporting parameter selection. One of the problems left open is in fact the selection of parameters at the beginning of the analysis, when the statisticians have no indication which parameter(s) to select first. This solution considers the domain knowledge, to directly suggest possible parameter combinations, depending on the data domain. By exploiting the metadata and the provenance information of the input signals, it is usually possible to automatically trace the data back to a specific domain (Q8). In such a way, besides the orienting support that comes from the integration of hints in the sliders, the statisticians can be directly guided towards parameters that make more sense in certain scenarios. Such guidance can be framed as **directing** support (Q6) and it was appreciated by the statisticians especially to initiate the analysis.

Once the parameters have been chosen, the system launches the BSS algorithms which on average may take some seconds to produce the output, that is, the signals composing the original measurements. The second part of the workflow is dedicated to compare, explore and interpret the obtained results. The analysis cycle can then be repeated and better parameters be chosen.

The last operation the designer aims to support by means of guidance is the interpretation and comparison of the obtained results. At this point of the analysis, analysts must discern whether the results obtained make sense, also in light of the previous parameters combination and different output statistics. To support this task, the guidance mechanism makes use of a machine learning algorithm (the random forest algorithm is used) (Q9). It automatically classifies, groups together and presents to the users the results, which are usually composed of an unordered set of components. This is visualized in Figure 7b (Q8). Thanks to this support, the analyst is immediately presented with a reasonable classification of the results that helps him/her to interpret the output. At the same time, the designer provided interaction facilities and visualizations (superimposition of outcomes of different runs as well as the parameters associated with them) which allow the statisticians to easily compare the obtained signals with those of previous runs. To this end, the system stores the history of interaction and output results. This support can be framed as directing guidance (Q6).

**Step 4: Guidance feedback loop**. The last part of the design dealt with the definition of feedback mechanisms to let the statisticians steer the guidance process. The described mechanisms make assumptions, for example, on the domain and on the results classification, that the analyst might need to fine tune and refine. Therefore, when providing guidance, the designer also decided to offer means to modify parameters of the guidance. In general, the system stores the produced results and reuses them for future analysis. For instance, if the analysis reaches a positive conclusion, the results of the classification algorithms are added to the knowledge base to improve the classification algorithms. The same happens when a correct data domain is selected and proper parameters are suggested in the first phase of the analysis. All these small details add to the support of adaptive and trustworthy guidance (R3–R4).

The design took about one month and a half. For the sake of clarity, we described the resulting design in a linear fashion, although, in this temporal period, the designer iterated many times over all the steps and produced many design alternatives which were presented to the statisticians. The statisticians appreciated some ideas, rejected others which were not in line with their analysis workflow, and in the end settled for the design we described earlier. Finally, we asked the designer to pay attention to possible flaws and gaps in the guidance design framework, but no major issues were raised during the design. This shows how the framework considers carefully all major aspects involved in the design of guidance, and can be considered rather complete in this respect. As it has been written, the design of guidance poses many challenges and requires designers to foresee issues that may arise during the analysis. Our framework helps in this respect as it points designers to consider thoroughly all these aspects in a step‐wise process.

This section laid the basis for the evaluation and discussion of the benefits of our framework and showed how the design of guidance could be easily integrated in the VA process. In the following section, we summarize our observations.

## 6 Discussion

*Completeness of the framework*. When creating the framework for guidance designers, we took care that all important aspects needed for designing appropriate guidance are included. We cannot guarantee, however, that the output of the design process is complete. One important point we want to raise is, in fact, that our framework requires designers to think carefully, consider, and *foresee* all possible issues and problems that might occur during the analysis, and subsequently think of possible guidance solutions to overcome such situations. This can require a lot of effort from designers, which might make the design difficult to complete. One possible solution to this problem would be the implementation of learning mechanisms so that the guidance system can learn and improve over time, as new knowledge gaps arise.

*Design feedback and evaluation*. We see the design of guidance as a finite sequence of (reiterated) steps. This iterative process allows designers to keep design risks low. Still, the proposed framework is not an algorithm that can be applied automatically and not a formal procedure that can be followed thoughtlessly. As discussed previously, we see no practical way to guarantee in advance that the output of the process is complete with respect to the analysts' needs. We discuss this risk, as well as others, and show how to minimize the possible risks in Section 4.3.5. However, like with any design framework, proper evaluation of the design can be done only after the implementation of the designed system. What we can support, with our framework, is the consideration of the major design aspects that concur to an effective guidance solution.

The output of the design process could be evaluated in practical use‐case scenarios, where the analyst is faced with real analysis problems, thus testing the effectiveness of the designed guidance. Since this evaluation is taking place in a different time frame in respect to the design process, we consider it separate from the design itself. Hence, we did not include it explicitly in our framework.

*Integrating guidance and VA design*. We envision our guidance design framework to become an integral part of any VA design process. Our framework already integrates references to VA design in Step 1. However, it is still unclear how a tight integration can be accomplished. We see this as an important question for future research.

*Methodology and design procedure*. We point out that instead of exploiting a single source to build this framework, we derived best practice of guidance design from multiple sources. Our design framework is based on a careful analysis of existing VA process models and a characterization of guidance functions in related work. Moreover, we enriched our findings by our own research in guidance for applied VA methods. Thus, our design framework represents an integrated best practice of methods, and desirable properties for effective guidance. We believe that this framework will help researchers and practitioners in VA to approach the design of guidance solutions step by step and to consider critical aspects that are easily overlooked otherwise.

## 7 Conclusion and Future Challenges

In this work, we present a design framework and a set of qualitative requirements to guide the design of effective guidance in VA. Conversely to previous research that focused on describing the characteristics of the *process of guiding* [CGM19], our goal has been to describe the *process of designing guidance* (from the designers' perspectives) and to present it as a sequence of steps applicable to a wide variety of analysis scenarios.

To show the usefulness of our framework, we demonstrate its application to the design of three guidance approaches in different application domains and a design walk‐through in the domain of statistics. We list the challenges emerging in such scenarios and report how the framework can be used to design guidance solutions to mitigate them.

Finally, although we propose a comprehensive framework to design effective guidance, there are a number of challenges that remain unsolved:

C1‐ *How can we know/evaluate that guidance is effective?* Guidance should be considered effective, if it can solve the knowledge gap of the analyst. A qualitative study with the actual end users of a guidance‐enriched VA approach might be a suitable means to shed light on this aspect and should be an integral part of any evaluation of guidance methods.
C2‐ *How can a knowledge gap be inferred by the system?* We propose two general mechanisms, direct and indirect. However, these are rather abstract. Since guidance requires a context‐dependent solution, there is no general answer to this question. A combination of user modelling and the integration of expert knowledge seems promising.
C3‐ *How can a knowledge gap be conveyed to the system?* The research on mechanisms for the analyst to communicate knowledge gaps is still far away from providing a definite answer to this question. This involves finding ways to encode knowledge and communicate it effectively to a computational system.
C4‐ *How can we infer what degree of guidance is needed?* What degree of guidance is needed depends on the user knowledge, the tasks, and possibly the users' behaviour. Again, there is a need for further research in this direction. Similar to the inference of the knowledge gaps, a combination of user modelling and expert knowledge might help in this respect.
C5‐ *What methods exist to generate guidance?* An answer to this question requires the consideration of the task and scenario‐specific aspects. In our design scenarios, methods to generate guidance were chosen to reduce design risks, maximize effectiveness and support a seamless integration of guidance in the analysis process.
C6‐ *How can we decide the right moment for guidance?* Being on time is necessary for guidance to be effective. A designer needs to understand the scenario to understand when guidance should be provided: right away, after detecting a knowledge gap or even at a different point in time. It mainly depends on the how critical is the decision the analyst has to take.
